# The complete mitochondrial genome of an ancient cattle (*Bos taurus*) from Taosi site, China, and its phylogenetic assessment

**DOI:** 10.1080/23802359.2022.2073834

**Published:** 2022-05-10

**Authors:** Xing Zhang, Liu Yang, Xingbo Zhao, Hai Xiang

**Affiliations:** a Guangdong Provincial Key Laboratory of Animal Molecular Design and Precise Breeding, School of Life Science and Engineering, Foshan University, Foshan, China; b National Engineering Laboratory for Animal Breeding, Key Laboratory of Animal Genetics, Breeding and Reproduction, Ministry of Agriculture and Rural Affairs, College of Animal Science and Technology, China Agricultural University, Beijing, China

**Keywords:** Ancient DNA, cattle, Yellow River region, mitogenome, phylogenetic analysis

## Abstract

The complete mitochondrial genome (mitogenome) of one 4000-years-old cattle from Taosi site was determined by high throughput sequencing. The mitogenome was 16,336 bp in length and contained 13 protein-coding genes, two rRNA genes, and 22 tRNA genes. The protein-coding genes had two types of start codons (ATG and ATA) and three types of stop codons (TAA, TAG, and AGA). The overall base composition of the genome was 33%-A, 27%-T, 26%-C, 14%-G. The matrilineal genealogical analysis based on mitogenome revealed that the 4000-years-old cattle from Taosi site was domestic taurine cattle. In this study, we not only reported a complete mitogenome for a 4000-years-old bovine remain from the middle Yellow River region but also provided the mitogenomic evidence for the close phylogenetic relationship between the early taurine cattle in Northern China and modern domestic cattle.

Taurine cattle (*Bos taurus* Linnaeus, 1758), as one of the most important livestock, have been domesticated and utilized for thousands of years. It is widely accepted that taurine cattle are domesticated from wild aurochs in Near East (Felius et al. 2014; Verdugo et al. 2019). The Chinese taurine cattle is proposed to be introduced from Near East 5000–4000 years ago (Cai et al. 2014; Chen et al. 2018). Taosi, a 4000-years-old archaeological site in the middle Yellow River region, has been deemed as an important site representing the early culture of ancient China (Brunson et al. 2016). It is located between Ta'er Mountain and Fen River, and about 7.5 km to the northeast of Xiangfen County, Shanxi Provinces. Archaeological evidence suggest that the bovine remains at Taosi site are from the domesticated taurine cattle. However, it lacks genetic evidence to show their phylogenetic relationship to modern domestic cattle.

In this study, an ancient bovine phalanx remain was collected from Taosi site (35°52'N, 111°30′), which was dated back to 4350–3900 before the present (BP) based on radiocarbon dating. The specimen was deposited at China Agricultural University (www.cau.edu.cn, contact person: Xingbo Zhao, Email: zhxb@cau.edu.cn) and was labeled with archaeological ID 02JXTIhT5126H39③ and lab code TS1C. Since no animals were involved in this work, the ethical approval had been confirmed not applicable in this study. All ancient DNA extraction and library construction work were conducted at the Ancient DNA Laboratory of Foshan University. The adhering soils and other external contaminations of the bone were cautiously cleaned using abrasive paper, and then were washed with 5% (vol/vol) sodium hypochlorite solution and followed by double-distilled water and drying under UV irradiation. After that, the sample was ground to powder using an automatic sample quick grinding machine (Shanghaijingxin, China). Ancient DNA was extracted using Dabney's method (Dabney et al. 2013) and submitted to double-stranded DNA library preparation with VAHTS Universal Plus DNA Library Prep Kit for MGI (Vazyme, China) following the manufactory protocol. Then the qualified library was 100 bp paired-end sequenced on the BGISEQ500 platform. The raw data were quality assessed using FastQC v0.11.9 (https://www.bioinformatics.babraham.ac.uk/projects/fastqc/). A total of 952,643,896 paired-end reads were generated for TS1C. The obtained reads were processed through the EAGER v2.1.0 pipeline (Fellows Yates et al. 2021). Briefly, adapter and low-quality reads were filtered using AdapterRemoval v2.3.1 (Schubert et al. 2016) with parameters: –minlength 10 –minquality 20. Then, the retained clean reads were mapped to the reference mitogenome sequence (GenBank accession No.: V00654) using Circularmapper v1.0 (Peltzer et al. 2016) with parameters as -n 0.04 -l 1024. The resulting file was converted to bam file and then was sorted and filtered using Samtools v1.9 (Li 2011). Duplications were removed using Dedup v0.12.5 (Srinivasan et al. 2012). The endogenous DNA content, coverage rate, and mapping quality distribution were calculated using Qualimap v2.2.2-dev (Okonechnikov et al. 2016). And SNP calling was conducted using BCFtools v1.8 (Li 2011). The consensus mitogenome of TS1C was assembled and double checked manually and was subsequently annotated using MITOS2 webserver (Bernt et al. 2013). The complete mitogenome sequence of TS1C was deposited at GenBank with accession number MW364778.1.

As a result, the complete mitogenome of TS1C was 16,336 bp in length. The overall base composition of the genome was 33%-A, 27%-T, 26%-C, and 14%-G. It contained 13 protein-coding genes, two rRNA genes, and 22 tRNA genes. Among them, 28 genes were on the heavy strand while the rest nine genes were on the light strand. The 13 protein-coding genes had two types of start codons (ATG and ATA) and three types of stop codons (TAA, TAG, and AGA). Specifically, nine genes including *ND1*, *COX1*, *COX2*, *ATP8*, *ATP6*, *COX3*, *ND4L*, *ND6,* and *CYTB*, used ATG as the start codon while four genes including *ND2*, *ND3*, *ND4,* and *ND5*, used ATA as the start codon. *ND2* and *ND3* were ended with TAG while *CYTB* was ended with AGA, and the rest 10 protein-coding genes were ended with TAA as the stop codon.

The mitogenome of TS1C had nine SNPs to the reference sequence (V00654). To determine the genetic relationship of TS1C to other extant Bovidae, a Bayesian phylogenetic analysis was performed using TS1C mitogenome and 24 extant Bovidae mitogenomes from GenBank, including four *Bison* species, two *Bubalus* species, and eight *Bos* species. The Bayesian tree was constructed with MrBayes 3.2.7 (Ronquist et al. 2012) using GTR + I+G model which was identified by jModelTest 2.1.1 (Darriba et al. 2012). The consensus tree was depicted using FigTree v1.4.2 (http://tree.bio.ed.ac.uk/software/figtree/). The result showed that TS1C was closely relative to modern *Bos taurus* with a high bootstrap value (Figure 1), suggesting TS1C as a potential domestic taurine cattle rather than any other bovine. In summary, this study not only obtained a complete mitogenome for a 4000-years-old bovine remain from the middle Yellow River region but also provided the mitogenomic evidence for the close phylogenetic relationship between the early taurine cattle in Taosi site and modern domestic cattle.

**Figure 1.**
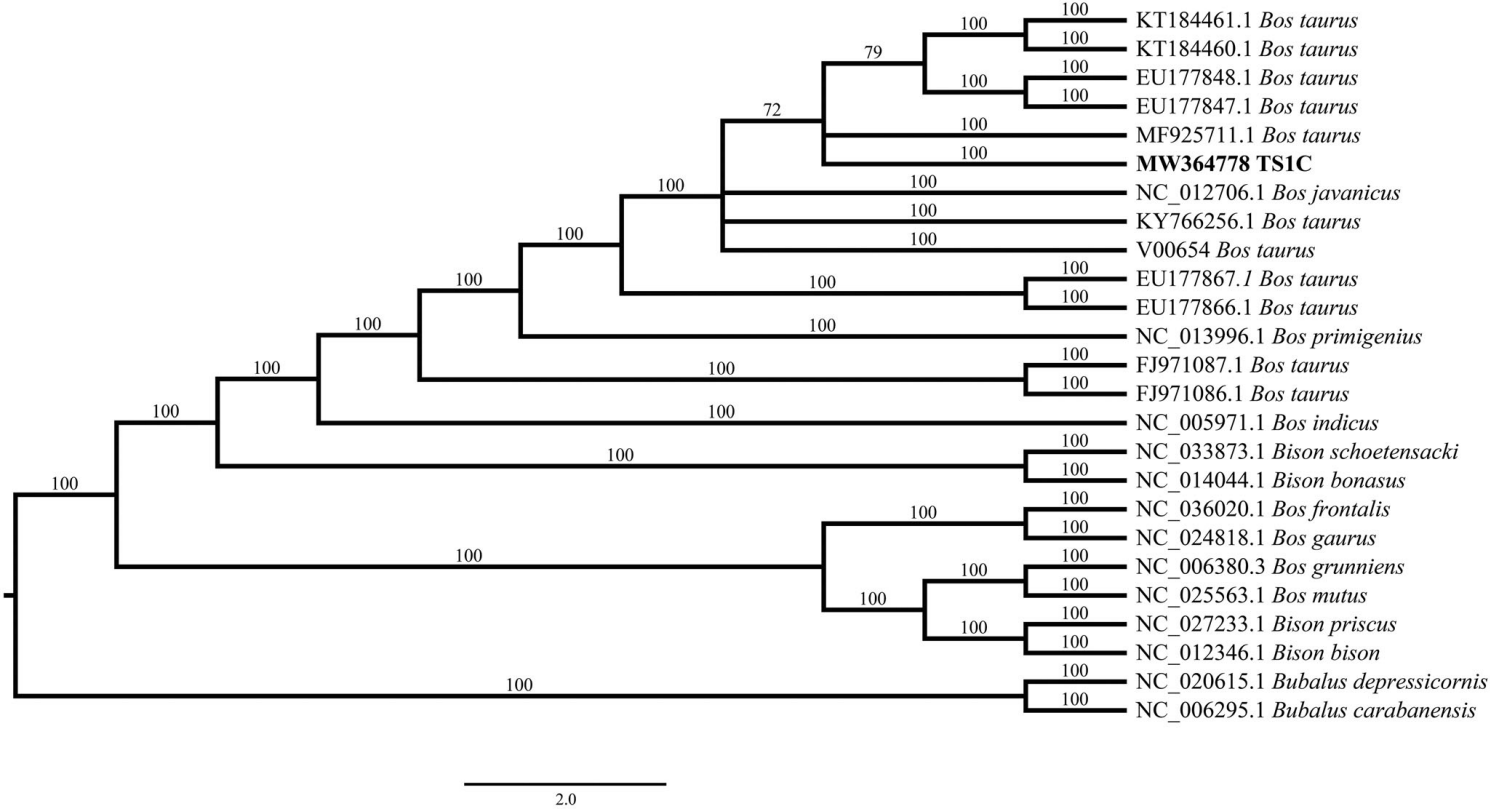
Phylogenetic tree based on the complete mitogenome of TS1C and 24 extant Bovidae mitogenomes.

## Data Availability

The mitochondrial genome sequence data that support the findings of this study are openly available in GenBank of NCBI at [https://www.ncbi.nlm.nih.gov] (https://www.ncbi.nlm.nih.gov/) under the accession no. MW364778.1. The associated BioProject, SRA, and Bio-Sample numbers are PRJNA773626, SRR16530732, and SAMN22502716 respectively.
